# Perivascular spaces as a marker of psychological trauma in depression: A 7‐Tesla MRI study

**DOI:** 10.1002/brb3.2598

**Published:** 2022-06-07

**Authors:** Daniel L Ranti, Andrew J Warburton, John W Rutland, Jonathan T. Dullea, Matthew Markowitz, Derek A Smith, Sophie Z Karwoska Kligler, Sarah Rutter, Mackenzie Langan, Annie Arrighi‐Allisan, Ilena George, Gaurav Verma, James W Murrough, Bradley N Delman, Priti Balchandani, Laurel S Morris

**Affiliations:** ^1^ BioMedical Engineering and Imaging Institute Icahn School of Medicine at Mount Sinai New York NY USA; ^2^ Department of Neurology, Massachusetts General Hospital Boston MA USA; ^3^ Department of Psychiatry Department of Neurology at the Mount Sinai Hospital New York NY USA; ^4^ Department of Diagnostic Molecular and Interventional Radiology Icahn School of Medicine at Mount Sinai New York NY USA

**Keywords:** blood–brain barrier, glymphatic system, major depressive disorder, medical imaging, MRI, psychological trauma

## Abstract

**Introduction:**

Emerging evidence in depression suggests that blood–brain barrier (BBB) breakdown and elevated inflammatory cytokines in states of persistent stress or trauma may contribute to the development of symptoms. Signal‐to‐noise ratio afforded by ultra‐high field MRI may aid in the detection of maladaptations of the glymphatic system related to BBB integrity that may not be visualized at lower field strengths.

**Methods:**

We investigated the link between glymphatic neuroanatomy via perivascular spaces (PVS) and trauma experience in patients with major depressive disorder (MDD) and in healthy controls using 7‐Tesla MRI and a semi‐automated segmentation algorithm.

**Results:**

After controlling for age and gender, the number of traumatic events was correlated with total PVS volume in MDD patients (*r* = 0.50, *p* = .028) and the overall population (*r* = 0.34, *p* = .024). The number of traumatic events eliciting horror was positively correlated with total PVS volume in MDD patients (*r* = 0.50, *p* = .030) and the overall population (*r* = 0.32, *p* = .023). Age correlated positively with PVS count, PVS total volume, and PVS density in all participants (*r* > 0.35, *p* < .01).

**Conclusions:**

These results suggest a relationship between glymphatic dysfunction related to BBB integrity and psychological trauma, and that glymphatic impairment may play a role in trauma‐related symptomatology.

## Graphical abstract

Emerging evidence in depression suggests that blood–brain barrier (BBB) breakdown and elevated inflammatory cytokines in states of persistent stress or trauma may contribute to the development of symptoms. We investigated the link between glymphatic neuroanatomy via perivascular spaces (PVS) and trauma experience in patients with major depressive disorder (MDD) and in healthy controls using 7‐Tesla MRI and a semi‐automated segmentation algorithm. We found an association between experience with traumatic events and PVS volume suggesting an association between psychological trauma and glymphatic dysfunction.

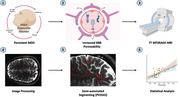

## 1 INTRODUCTION

Major depressive disorder (MDD) is a debilitating psychiatric condition with significant morbidity and is one of the most prevalent causes of disability across the world. MDD has an estimated global prevalence of 4.4%, or 330 million people as of 2015 (Conwell et al., 1996; Organization & Others, 2017). Despite ongoing research efforts, the pathophysiology of MDD is incompletely understood. Recent research, however, has begun to investigate novel biomarkers of depression, including increased peripheral immunologic profiles and central blood–brain barrier (BBB) breakdown (Copeland et al., 2012; Haastrup et al., 2014; Kennis et al., 2020; Khandaker et al., 2014; Pasco et al., 2010). Mechanistically, chronic social stress in rodent models has been associated with the downregulation of BBB tight junctions, reducing BBB integrity and allowing the permeation of cytokines such as interleukin‐6 (IL‐6) into the brain, which coincides with the development of depressive behaviors (Menard et al., 2017). In humans, psychological trauma has similarly been associated with dysregulation of the inflammatory system, predisposing patients to depression (Baumeister et al., 2016; Baumeister et al., 2014; Dantzer et al., 2008).

With recent advances in magnetic resonance imaging (MRI) field strength, neurobiological theories related to BBB integrity can now be examined in human patients with depression in vivo (Brown et al., 2019; Menard et al., 2017). Of particular interest is the role of the integrity of small perivascular spaces (PVSs), which form around neural vasculature and play a role in the clearance of chronic inflammatory mediators in the brain (Rasmussen et al., 2018; Rudie et al., 2018). The brain‐wide network of PVSs create a metabolic waste drainage system, called the glymphatic system. The number of PVS spaces has been shown in association with reduced integrity of the BBB in the basal ganglia (Li et al., 2019). Glymphatic malfunction has been implicated in the disease progression of a number of neurocognitive processes, including aging, stroke, Alzheimer's, and migraine (Gaberel et al., 2014; Kress et al., 2014; Peng et al., 2016; Schain et al., 2017); however, no studies have yet investigated the link between PVS neuroanatomy and psychological trauma in depression in vivo via the use of ultra‐high field strength MRI.

Prior to the development of 7T imaging, MRI techniques at lower field strengths were more limited in their ability to depict small‐scale structures, such as PVSs, with sufficient resolution and sensitivity. In direct comparisons between field strengths, 7T scanners have been shown to provide superior signal‐to‐noise ratio (SNR) and resolution than is achievable at 3T (Barisano et al., 2021). For example, when identifying similar small‐scale anatomical changes in multiple sclerosis (MS) patients, a central vessel was successfully identified in 87% of lesions at 7T versus 45% at 3T (Tallantyre et al., 2009). Thus, accurate studies quantifying PVS anatomy have only recently been made possible with the advancement of high‐resolution ultra‐high field strength MRI. The aim of this study was to leverage technological advances in MRI field strength to investigate the associations between psychological trauma and whole‐brain PVS quantification in patients with MDD.

## 2 METHODS

### 2.1 Setting and participants

Following Icahn School of Medicine at Mount Sinai (ISMMS) Institutional Review Board approval, MDD and healthy control (HC) participants were recruited through the Depression and Anxiety Center at ISMMS. All participants recruited were between 18 and 65 years of age, were English speaking, and had either a current primary diagnosis of MDD (MDD group), or no psychiatric diagnoses (HC group), as assessed by either the Structured Clinical Interview for DSM‐IV Axis I Disorders (SCID) or the Structured Clinical Interview for DSM‐5 Research Version (First et al., 2015; *Structured Clinical Interview for DSM‐IV Axis I Disorders: Patient Edition (February 1996 Final), SCID‐I/P*, 1998). All patients gave fully informed written consent prior to inclusion in the study.

Patients with comorbid anxiety disorders, with the exception of obsessive‐compulsive disorder (OCD), were included in the MDD group. Exclusion criteria for the MDD group included any history of substance use disorder, psychotic disorder, bipolar disorder, pervasive developmental disorder, intellectual disability, or current diagnosis of OCD or MDD with psychotic features. Exclusion criteria for the HC group included any diagnosis of any psychiatric conditions. In both groups, participants were excluded if they had an unstable medical illness, were at serious risk for suicidal behavior (as determined by a study psychiatrist), had any contraindications for MRI (including pacemakers or metallic objects in the body), or were pregnant, nursing, or by self‐report could become pregnant during the course of the study.

### 2.2 Clinical measurements

As part of screening for the study prior to MRI data acquisition, each patient was interviewed using the SCID and Montgomery‐Asberg Depression Rating Scale (MADRS) and was asked to complete a number of self‐reported measures. The SCID provided information about the duration, onset, and number of depressive episodes of MDD. The MADRS provided information about specific symptoms domains within MDD, as well as a total severity score (Montgomery & Asberg, 1979). The self‐report measures included the Traumatic Life Events Questionnaire (TLEQ), which assesses the frequency and severity of adult traumatic experiences throughout the lifespan. The TLEQ provides three subscales: a count of traumatic events experienced (CE), a count of traumatic events that elicited fear, helplessness, or horror (OC), and a total frequency of events (Kubany et al., 2000). Self‐report measures were also assessed in the HCs.

### 2.3 MRI sequences

**FIGURE 1:**
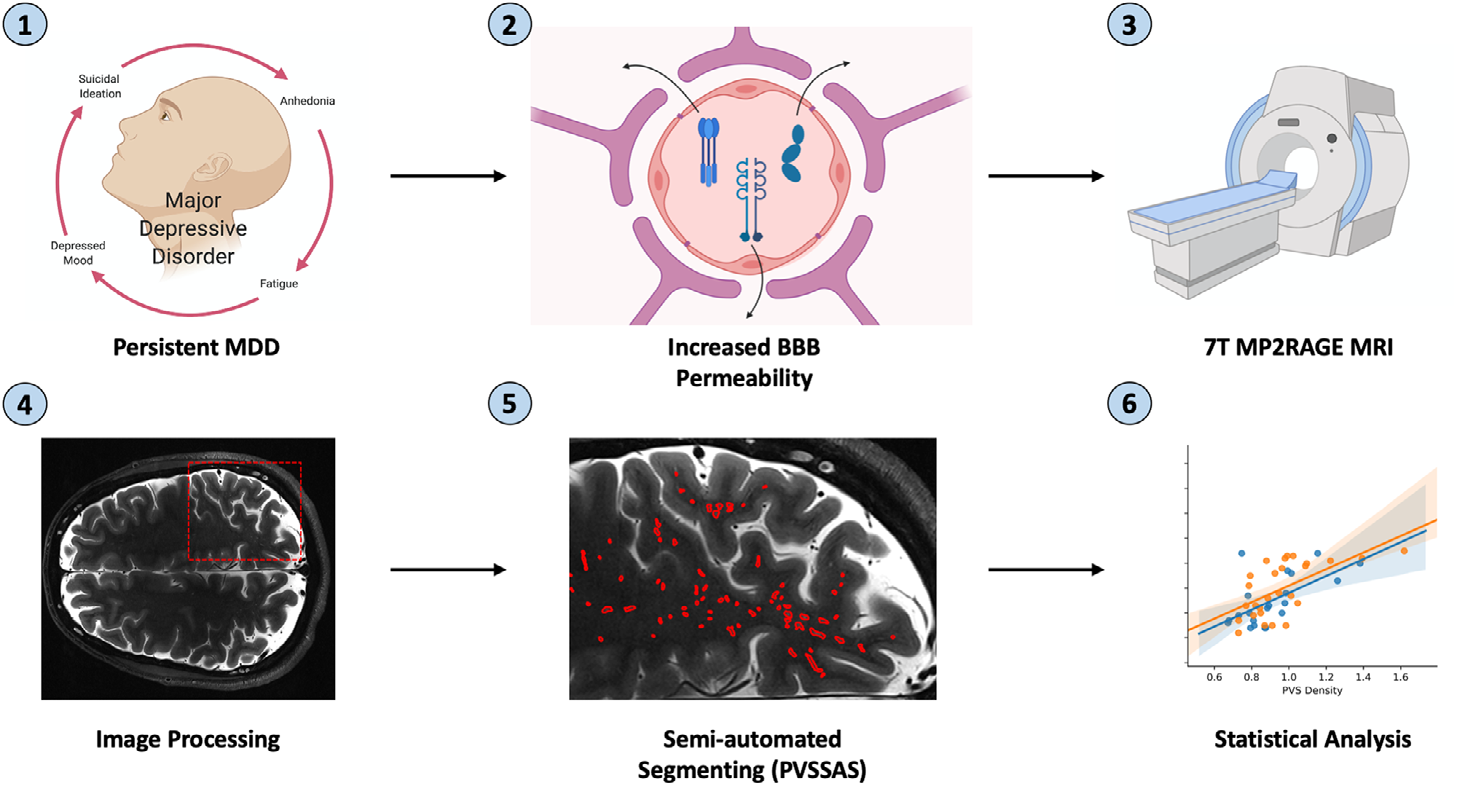
Project workflow. An outline of the project workflow from scan acquisition to final statistical analyses. (1) Persistent MDD symptoms lead to (2) the breakdown of the blood brain barrier via inflammatory cytokines such as IL‐1, IL‐6, and TNF‐α; (3) 7T MRI scans along with whit matter mask data were collected on a Siemens 7T whole body scanner; (4) the white matter data were coregistered into the T2‐space using FreeSurfer; (5) semi‐automated PVS markings were generated using a Frangi‐based edge detection filter; (6) statistical analyses were run correlating various trauma scores to PVS quantification measures

In order to measure PVSs, patients underwent axial T2‐weighted turbo spin echo (TSE) scan sequences and T1‐weighted MP2RAGE sequences on a 7‐Tesla whole body MRI scanner (Magnetom; Siemens Healthcare, Erlangen, Germany) (see Figure 1). The T2‐TSE scan sequence was chosen based on prior studies highlighting the high contrast‐to‐noise ratio in visualizing PVS morphology, and proven efficacy in visualizing PVSs in epilepsy patients (Feldman et al., 2018; Zong et al., 2016). The T2‐TSE scans generated a total of 54 slices per patient. The repetition time was 9000 ms and echo time was 59 ms. The acquired voxel resolution was 0.4 × 0.4 mm in‐plane interpolated to 0.2 × 0.2 mm with 2 mm slice thickness and a 0.6 mm gap between slices, the acquisition matrix dimensions were 512 × 432 mm with a final interpolated resolution of 1024 × 864, and the field of view was 200 × 169 mm. The orientation of the scan was axial.

White matter masks (WMMs) were generated using the T1‐weighted MP2RAGE sequence. This segmentation was performed using the FreeSurfer Atlas image analysis suite version 6.0 (http://surfer.nmr.mgh.harvard.edu). The number of slices for a single slab was 240. The repetition time was 6000 ms and the echo time was 3.62 ms. The voxel resolution was 0.7 mm^3^ isotropic, the acquisition matrix was 320 × 260 mm, and the field of view was 225 × 183 mm. The orientation of the scan was coronal. The T2‐TSE scans were also processed using FreeSurfer with nonparametric nonuniform intensity correction, intensity normalization, skull stripping and neck removal, automatic segmentation, and parcellation processing steps. The T1‐weighted WMM was then coregistered to the T2 space using the FreeSurfer Atlas program. Total brain and CSV volumes were also calculated using FreeSurfer segmentation.

### 2.4 Semi‐automated, Frangi‐filter segmentation algorithm

**FIGURE 2:**
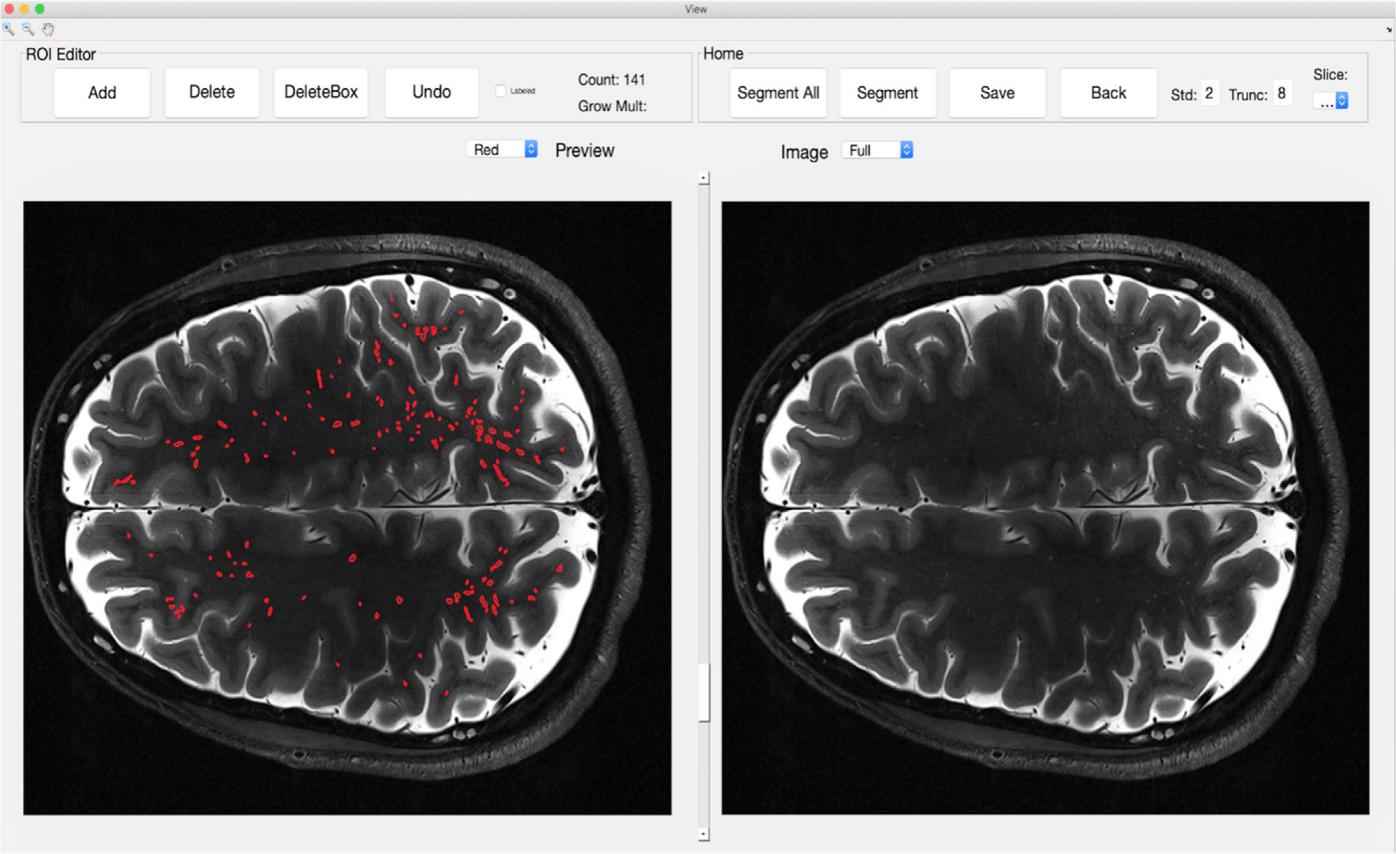
Unsegmented and segmented 7T MRI Scan. A side‐by‐side comparison of a segmented axial T2‐TSE slice (left) and the unsegmented image (right) in the PVS‐SAS program. Each red outlined object on the marked slice (left) delineates a PVS, as detected by the semi‐automated PVS marking, and confirmed by two manual readers

In order to delineate the PVSs on each scan slice, the customized Perivascular Space Semi‐Automatic Segmentation (PVS‐SAS) algorithm was created. PVS‐SAS is a semi‐automated tool for segmenting, viewing, and editing PVS data on MRI slices using a Frangi filter‐based edge detection algorithm. Frangi filtering is a rapid, powerful technique to segment vessel‐shaped objects in two and three dimensions (Frangi et al., 1998). Frangi filters have been successful in segmenting PVSs, showing strong correlations between single‐slice PVS count and total volume when compared with manual raters (Ballerini et al., 2018). The following filter parameters were used: scale range = 1.4–3.2, scale threshold = 2, *β*_1_ = 0.95, and *β*_2_ = 0.35. These are the MATLAB default parameters. By employing a Frangi filter, we sought to minimize interobserver variability and fatigue in segmenting MRI scan data, and to reduce the time required to detect the PVSs. PVS‐SAS is run within the MATLAB integrated development environment and is created using MATLAB's built‐in graphical user interface (GUI) editor, GUIDE (see Figure 2). A GUI‐based mechanism was chosen to allow for a user to add, delete, and change the markings on a given slice without prior knowledge of MATLAB or programming, providing high clinical translation. PVS‐SAS employs several key steps to segment MRI data, including preprocessing, Frangi filter‐based segmentation, post‐processing, and manual editing. First, PVS‐SAS preprocesses the WMMs by filling in small holes (<200 pixels), using a gaussian filter with a standard deviation of 1. This step addresses potential partial volume limitations, whereby the FreeSurfer segmentation engine includes tiny regions of gray matter entirely surrounded by white matter. Next, PVS‐SAS runs the Frangi filter to identify likely PVSs.

Following detection with the Frangi filter, PVS‐SAS removes boundary pixels (default value set at 8 pixels) to exclude false positives along the boundary of the WMM. In postprocessing, PVS‐SAS thresholds the resulting pixel intensities (default pixel intensity of 0.4), and removes objects identified by the Frangi filter that are not within a certain range (default value of 2 standard deviations from mean pixel intensity). These parameters used in the Frangi filter settings were based on optimal clinical parameters as determined by the existing literature (Ballerini et al., 2018). Following the full processing of each slice, the GUI then allows the user to quickly review and alter the automated markings to remove false positives, or add markings if any obvious missed PVSs are identified upon manual inspection.

Two separate readers performed the semi‐automated markings. Readers were trained to identify PVSs by a Certificate of Added Qualification neuroradiologist with more than 20 years of experience. The readers then used PVS‐SAS to automatically mark PVSs within the segmentations, removing marked objects that were not PVSs, but adding no additional markings. Interrater reliability on a single subject was assessed by calculating the percent overlap of PVSs that were in the identical voxel‐for‐voxel location between the two readers on a single subject and by the Dice score. Based on the results of this analysis, and the highly automated nature of the PVS‐SAS program in which only a small fraction of the marked PVSs were altered, each reader segmented a non‐overlapping half of the remainder of the patients.

### 2.5 PVS‐SAS overlap analysis

The PVS‐SAS method was evaluated by sensitivity and specificity calculations as compared to manual markings. Time differences between manually generated tracings and PVS‐SAS generated tracings were gathered to understand the logistical impact of the tool. First, manual tracings were performed on a T2‐TSE scan from a single subject. The manual reader was a trained MD with experience marking and quantifying PVSs. The manual reader marked the PVS regions within the entire scan using OsiriX, manually labeling the cross section of every object deemed to be a PVS. The reader marked any point along the cross section, not necessarily in the geometric middle of the object. Next, PVS‐SAS was used by one of the trained semi‐automated readers to label the PVSs within the white matter of the same scan (see Figure S1). On review by two trained raters, objects that were mislabeled were removed, but no additional PVSs were added.

After the manual markings and the semi‐automated markings with PVS‐SAS had been generated, both scans were aligned and imported into MATLAB. In order to accurately compare the differences between the manual and automated scans, only PVSs marked within the white matter of each scan were considered. Furthermore, due to the intricate nature of PVS morphology, and the lack of easily applicable, predefined criteria, the manual readers and the semi‐automated program disagreed in certain instances on whether a given marking was a single PVS or multiple distinct PVSs. Given that each patient scan depicted thousands of PVSs, we made the assumption that each PVS marked, both in the manual and the semi‐automated markings, was a unique PVS. It is a time‐intensive and clinically involved process to determine whether or not two proximal PVS markings are indeed separate, or a single PVS that has traveled in and out of the plane of the MRI. For these reasons, PVSs on adjacent slices were also considered independently, as the distances between slices is 2.6 cm, and it would be inaccurate to assume PVSs on one slice are identical to markings in the slice prior.

Due to the differences in marking criteria, we used a bounding box analysis to determine the overlap between the manual and semi‐automated tracings. A bounding box was defined as the smallest two‐dimensional rectangle that entirely encircles a PVS marking generated by the manual or semiautomated procedure. We then counted any PVS in the two readings that overlapped with each other as being consistent with each other. In order to account for the inconsistencies in the methods between the manual and semi‐automated markings, as well as the intricacies of marking proximal PVSs, we allowed a tolerance of 4 mm to the edge of the bounding box.

### 2.6 Segmentation and statistical analysis

The collected MRI data were segmented using the semi‐automated Frangi‐filter method and reviewed by two independent readers. Following generation of the automated markings, each scan was manually inspected on a slice‐by‐slice basis for misclassification of PVSs due to white matter hyperintensity. A mismarked PVS was defined as any clearly mismarked hyperintense structure. Mismarked structures were primarily the borders between the white matter and the ventricles, sulci, and imaging artifacts near the apex and base of the skull. In each case of a mismarked PVS, the marking was removed. No further markings were added in addition to the automatically generated ones.

Following the semi‐automated segmentation, each scan was exported as a binarized .mat file, with voxels of 1 indicating areas corresponding to PVSs, and voxels of 0 denoting all other areas. The PVSs were then characterized on a two‐dimensional per‐brain basis, generating the following statistics for each subject: PVS density (PVS voxel count/white matter volume), PVS count, median PVS volume, total PVS volume, median equivalent diameter, median long axis length, and median short axis length. Prior to any statistical analyses, imbalances in gender distributions between the two were assessed with a chi square test (significance *p* < .05). Imbalances in age between the MDD and HC groups were evaluated with a two‐sided *T*‐test. A *t*‐test is a valid statistical test in this instance given that *n* > 30 thus the central limit theorem applies. Group‐wise differences between MDD patients and HCs in PVS measures were performed with a two‐sided independent *T*‐test.

Prior to any pairwise statistical correlations, the normality of each measurement was assessed using the Shapiro–Wilk test. Shapiro–Wilk was chosen to assess normality due to its performance in small sample sizes (Ghasemi & Zahediasl, 2012). In every case in which both distributions of the data being correlated proved to be normally distributed, parametric tests of correlation, namely Pearson correlation, were performed. In every instance in which one or both of the measures failed to prove normality, Spearman's nonparametric test of correlation was used (Kim, 2015; Puth et al., 2015). Pairwise statistical correlations between PVS measures and demographic or symptom severity scores were evaluated first using partial correlation coefficient tests without correction. For all statistically significant correlations, partial correlations were run with corrections for age, gender, brain segmentation volume without ventricles, and CSF volume (Stevens, 2012). All statistical analyses were performed with the R statistical computing language (R Foundation for Statistical Computing, Vienna, Austria).

## 3 RESULTS

### 3.1 Participants and descriptive data

In total, 48 subjects were enrolled in this study, including 27 HCs and 21 MDD patients. Demographic and clinical information are provided in Table 1.

**TABLE 1:** Participant demographics and accompanying statistics and scores

	MDD	Healthy controls	
*N*	21	27	*p* Value
Male gender	12	18	.707
Age (mean (SD))	34.95 (10.15)	39.70 (10.77)	.127
Total education			.059
Grade 7–12, no graduation	1	0	
Some college	4	4	
Graduated 2‐year college	1	3	
Graduated 4‐year college	7	6	
Some graduate/professional degree	6	2	
Graduated professional degree	2	12	
Average MDD duration (mean (SD))	73.53 (83.88)	N/A	
Age of MDD onset (mean (SD))	19.36 (9.42)	N/A	
Average MDD number of depressive episodes (mean (SD))	7.71 (12.26)	N/A	
MADRS (mean (SD))	30.14 (5.84)	0.50 (1.07)	<.001
CE TLEQ (mean (SD))	3.19 (3.39)	1.64 (1.62)	.039
OC TLEQ (mean (SD))	10.19 (12.60)	3.29 (4.13)	.009
Comorbidities	5.95 (2.89)	5.25 (1.00)	
Dysthymic disorder	14	0	
Social anxiety disorder	10	0	
Generalized anxiety disorder	5	0	
No comorbidities	5	27	

Group differences within the clusters were assessed for significance using a two‐sided T test for continuous variables, and a chi square test for categorical variables.

### 3.2 Semi‐automated PVS quantification

The semi‐automated PVS quantification method (PVS‐SAS) took approximately 25 min, compared with manual ratings, which took 6 h. The sensitivity and specificity of the semi‐automated method with a tolerance of 0.4 cm, as compared with the manual markings, were calculated to be 82.9 and 91.9%, respectively. There was strong inter‐rater reliability of the semi‐automated PVS quantification method (97.8–99.2%% full voxel‐for‐voxel overlap of the PVSs identified between two readers, Dice score = 0.9914. Dice score cannot be greater than 1).

### 3.3 Statistical analysis

**FIGURE 3:**
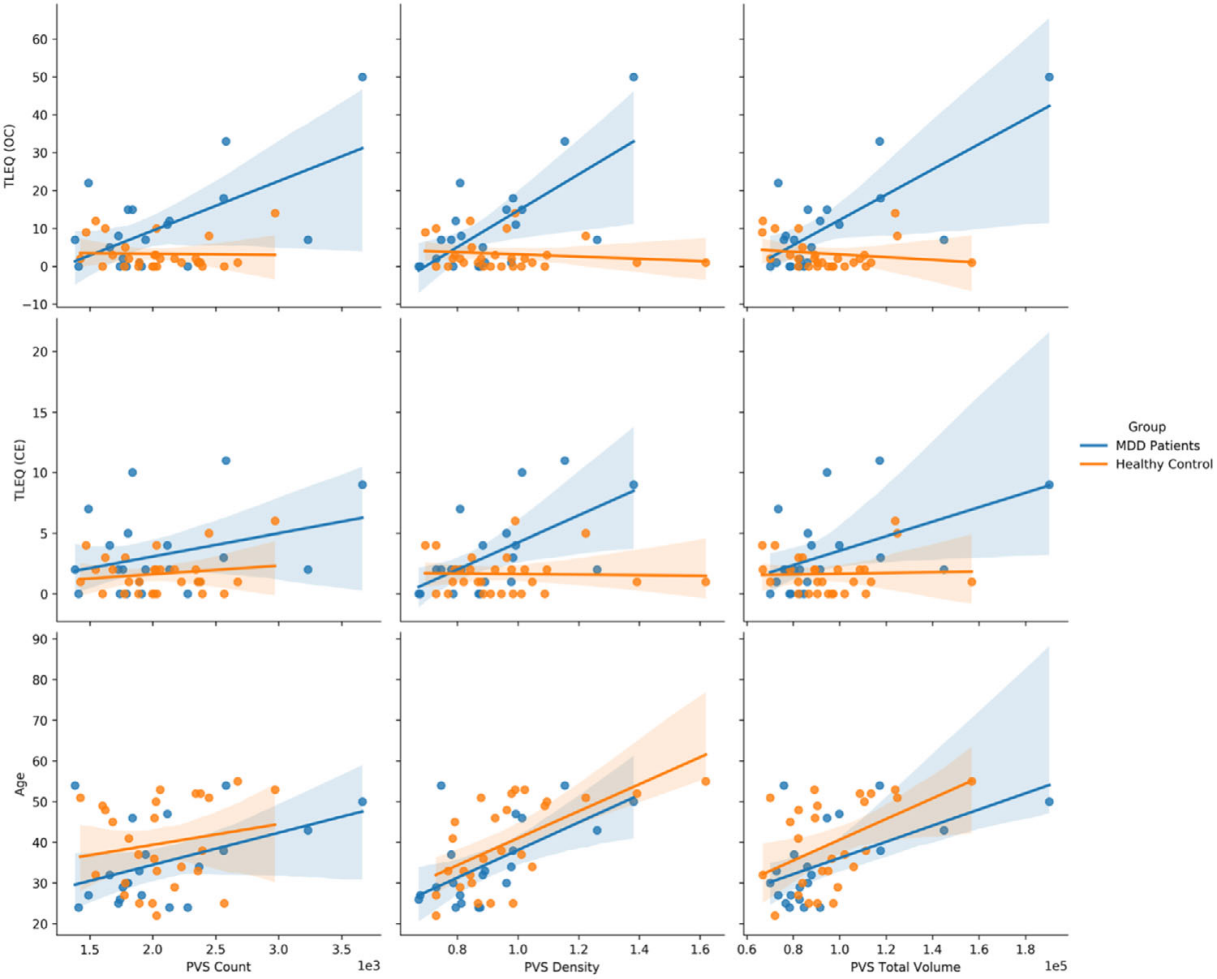
Scatter plots comparing OC TLEQ and age to PVS count, PVS density, and PVS total volume

Average PVS quantification measures for MDD patients as well as HCs are shown in Table 2. As expected, across the entire population, age correlated positively with PVS count (*r* = 0.37, *p* = .013), PVS total volume (*r* = 0.53, *p* < .001), and PVS density (*r* = 0.68, *p* < .001; see Figure 3). There were no statistically significant differences in PVS measures between MDD patients and HCs (Table 2). However, there were several statistically significant correlations between PVS measurements and lifetime trauma experience in MDD. In every test, one or both of the correlation measures failed to prove normality, and therefore Spearman's correlation coefficient was used in all pairwise correlates. After controlling for age and gender, total PVS volume was statistically and positively correlated with OC TLEQ scores (*r* = 0.50, *p* = .030) and CE TLEQ scores (*r* = 0.50, *p* = .028) in MDD patients. In addition, in all participants, total PVS volume was positively correlated with OC TLEQ (*r* = 0.32, *p* = .023) and CE TLEQ (*r* = 0.34, *p *= .024). OC and CE TLEQ correlations in MDD patients survived correction for CSF volume and brain segmentation volume (*p* < .05, *r* > 0.45). Significance was lost when correcting for CSF volume and brain segmentation volume in the entire population. MADRS score was not significantly correlated with PVS measures.

**TABLE 2:** PVS measures for both healthy controls and MDD patients

	MDD	HealthyControls	
*N*	21	27	*p* Value
Median PVS volume (mean (SD)), mm^3^	1.99 (0.19)	2.02 (0.13)	.568
PVS count (mean (SD))	2060.14 (572.02)	2041.82 (379.21)	.893
Total volume (mean (SD)), mm^3^	7517.99 (2276.72)	7627.83 (1582.4)	.843
Density (mean (SD)), PVS/mm^3^	11.38 (2.25)	11.88 (2.5)	.434
Median Eq. distance (mean (SD)), mm	0.72 (0.02)	0.73 (0.02)	.520
Median long axis (mean (SD)), mm	1.58 (0.12)	1.59 (0.07)	.658
Median short axis (mean (SD)), mm	0.84 (0.02)	0.84 (0.01)	.627
White matter volume (mean (SD)), dm^3^	0.49 (0.06)	0.46 (0.06)	.177

Reported values are means across patients of intrapatient median values.

## 4 DISCUSSION

Despite the widespread prevalence and debilitating consequences of MDD, current diagnostic criteria are reliant on subjective clinical features, and our understanding of the neuropathology of the disease remains limited. Trauma prevalence in MDD is high, supporting the hypothesis that traumatic psychological exposure induces a specific psychological phenotype of elevated stress and depressive symptoms (Flory & Yehuda, 2015; Rytwinski et al., 2013). This study aimed to contribute to the growing body of knowledge regarding glymphatics and the breakdown of the BBB in chronic stress by assessing PVSs as a biomarker for disease severity and progression. The key findings of this study indicate a relationship between traumatic psychological experience and increased PVS volume in MDD patients and HCs. In particular, after accounting for age and gender, the count of traumatic events that specifically elicited fear, helplessness, or horror, were positively correlated with the total PVS volume in the MDD population as well as the overall study population.

The results positively correlating PVS measures with the total number of traumatic events suggests that a prolonged state of stress, as is seen in MDD, may predispose patients to subtle physical alterations in their neuroanatomy. These changes may be driven by the breakdown of the BBB, reflecting a state of prolonged insult by proinflammatory cytokines. BBB breakdown and leakage has been shown in animal models and clinical studies to cause dilation of glymphatic structures as a result of the toxic insult (Rasmussen et al., 2018; Yu et al., 2019). Alterations in this system and the continued presence of a dilated state could predispose patients to ineffective clearance and maintenance of the neurological immune environment. It should be noted that present conclusions about links between BBB and PVS based on largely indirect measures. Future studies must measure PVS quantity/volume as well as BBB leakiness (as quantified with Gd‐DCE‐MRI or Arterial spin labeling ‐based techniques) in the same population to determine these relationships more empirically. Last, similar work should be done in translational model species with higher resolution MRI (e.g., 11.7T MRI in rats).

The link between psychological symptoms and PVS quantification is supported by existing studies that have explored the relationship between the glymphatic system and a variety of neurodegenerative diseases. Though MDD is not classically considered a neurodegenerative disease, its association with Alzheimer's disease (AD), Huntington's disease (HD), MS, and Parkinson's disease (PD) has nonetheless been well documented. Recent investigations of the pathophysiological and molecular mechanisms of MDD have revealed several similarities between the neurobiological changes contributing to MDD and the neurodegeneration present in AD, HD, MS, and PD (Galts et al., 2019). Furthermore, several monoclonal antibody therapies for MS, including natalizumab, have been approved by the United States Food and Drug Administration on the basis of reducing inflammation and leukocyte diapedesis across the BBB. 64.3% of MS patients treated with natalizumab remained free of clinically active symptoms, as compared with 38.9% of placebo treated patients (Havrdova et al., 2007; Rudick & Panzara, 2008).

While AD is a distinct disorder from MDD, numerous studies have indicated that MDD is a high‐risk factor for the development of AD. It is believed that damage to the glymphatic system induced by depressive disorder leads to impaired clearance of neuronal waste exchange and the accumulation of protein products in the brain (Xia et al., 2017). Supporting this notion, researchers determined that inducing depression in mice led to impaired functioning of the glymphatic clearance pathway and contributed to the deposition of exogenous human beta amyloid 42 (Aβ42) in the brain, ultimately resulting in neurodegeneration (Xia et al., 2017).

The association between white matter hyperintensities and small vessel disease and patients with depressive symptoms specifically has largely been explored in patients with post‐stroke dementia or late‐life depression. However, a number of lines of evidence in this patient population further support the theory that vascular malfunction leads to depressive symptoms. On a macrovascular scale, stroke patients are found to have a significant burden of depression, with 23.3% of patients in an outpatient pool found to have MDD (Robinson, 2003). On a microvascular scale, meta‐analysis of seven studies observing plasma markers of endothelial dysfunction saw statistically significant associations between soluble intercellular adhesion molecule‐1 and depressive symptoms (van Agtmaal et al., 2017). Furthermore, the homocysteine hypothesis of depression postulates that elevated levels of homocysteine can cause vascular malfunction, neurotransmitter alterations, and depressed mood (Folstein et al., 2007). Our observation that malfunction or stress of the glymphatic system, as defined by increased number of MRI‐visible PVS in patients with more severe depressive symptoms compared with HCs, fits with the above observations linking breakdown of microvascular architecture to depression.

Clinically, the results of this study suggest that, with further exploration, glymphatic quantification and visualization could be a useful tool to estimate the neurobiological impact of long‐term depression. Indeed, the results above indicate a statistically significant relationship between total trauma scores and PVS count and total volume in both MDD patients and HCs. Based on the results of the sensitivity and specificity analysis, the PVS‐SAS is a clinician‐friendly tool that achieves a high degree of accuracy and may obviate the time‐intensive and potentially error‐prone process of manually marking thousands of PVSs on MRI data. The PVS‐SAS program also experiences extremely high interrater reliability wits 97.8 and 99.2% identical overlap between the two semi‐automated readers, respectively. The inter‐reader reproducibility is a benefit of the semi‐automated method, though we are not able to do a direct quantitative comparison to prior literature reported values given differences in methodologies. In future work, we plan to future validate the use of this method against the manual method in a greater number of scans. This additional assessment would help ensure that the method is valid across factors such as age and comorbidity status. Future studies are warranted in order to assess the validity of these models in humans and to explore other, less well understood models of disease within MDD.

There are a number of limitations to this study as it was performed. The first of which is the relatively limited number of patients included in the analysis. While there were enough to characterize potential relationships, a larger sample size would provide better estimates for the effect size of the proposed relationships. Biomarkers of inflammatory markers, such as CRP levels, and medication status were not recorded as part of this study, and therefore we cannot control for other underlying reasons of blood brain barrier permeability. Although none of the patients in this study had been diagnosed with chronic inflammatory diseases, medication status and biomarkers of inflammation are important to consider when drawing conclusions regarding the correlation between depression and PVS morphology. Additionally, we did not collect data on vascular risk factors which also could feasibly be related to both depression and lymphatic dysfunction. Furthermore, the overlap analysis of the PVS method took place on manual markings, however the gold standard validation would be a comparison to histological markings. While brain biopsies for histological analysis were not available to us, further validation of this method would be useful on histological sections. Additionally, while each PVS was considered independently, we recognize the possibility of a single PVS traveling in‐ and out‐of‐plane thus artificially increasing the PVS count. It is also unlikely that a given PVS would cross slice planes given the relatively large slice thickness of 2 mm with a 0.6 mm gap. This consideration would impact manual and automated markings alike, however future work validating the findings of any two‐dimensional PVS analysis would benefit the field by focusing on quantifying distinct PVSs in‐plane. Another limitation is the relatively large slice thickness of 2 mm. This slice thickness was chosen to provide adequate coverage of the whole brain; however, this could lead to lower precision in the assessment of PVS volume as they move in and out of plane. As 7T MRI becomes increasingly available, quantification of small‐scale PVSs will become more common. However, high‐precision quantification of fine‐scale PVS morphology relies on the resolution best achieved at 7T, which enables high resolution while maintaining sufficient SNR to detect fine structures. The results of this study may not be fully reproducible on 3T machines because there is potentially insufficient SNR at the chosen resolution. To address the limitation of reproducibility at lower field strength, we limited the analysis to measures of PVS volume, density, and count. Measures of PVS size would heavily influence by the technique used and would systematically biased high for a study that utilized lower resolution. The trends and or counts observed at 3T may not concur with the results achieved at 7T, particularly if smaller PVSs are disproportionately represented and undercounted at 3T.

## 5 CONCLUSION

This study using ultra high‐field MRI imaging in MDD suggests that prevalence of PVSs is positively correlated with lifetime trauma events in patients with MDD. Specifically, after controlling for age and gender, we found statistically significant correlations between traumatic psychological experiences and the total PVS volume in MDD patients alone, as well as the entire study cohort. However, the difference in the whole cohort is likely driven by the difference seen in the MDD patients. These results suggest that PVS has potential as a useful biomarker in estimating symptom severity and its long‐term impact on neuroanatomical structures. Further studies would benefit from a prospective, longitudinal approach with a larger sample size.

## CONFLICT OF INTEREST STATEMENT

the authors have no conflicts to declare.

## FUNDING

Institutional funds from the Icahn School of medicine in Mount Sinai were used.

### PEER REVIEW

The peer review history for this article is available at https://publons.com/publon/10.1002/brb3.2598

## Supporting information


**Supplemental Figure 1: Semi‐automated Validation**. A single slice highlighting the manual markings (red objects) and semiautomated markings (green objects). The manual readers created each mark in OsiriX by marking the cross section of each object the readers deemed to be a PVS. In comparison, the semi‐automated program outlined the entire area of each object determined to be a likely PVS. Sensitivity and specificity for the semi‐automated method were 82.9% and 91.9% respectively, when compared to the manual markings.

## Data Availability

The data used will be made available upon request.
