# Supplementary figures and images for: Perivascular spaces as a marker of psychological trauma in depression: A 7‐Tesla MRI study
Source: Brain Behav. 2022 Jun 7;12(7):32598. doi: 10.1002/brb3.2598 (PMC9304831; doi:10.1002/brb3.2598)

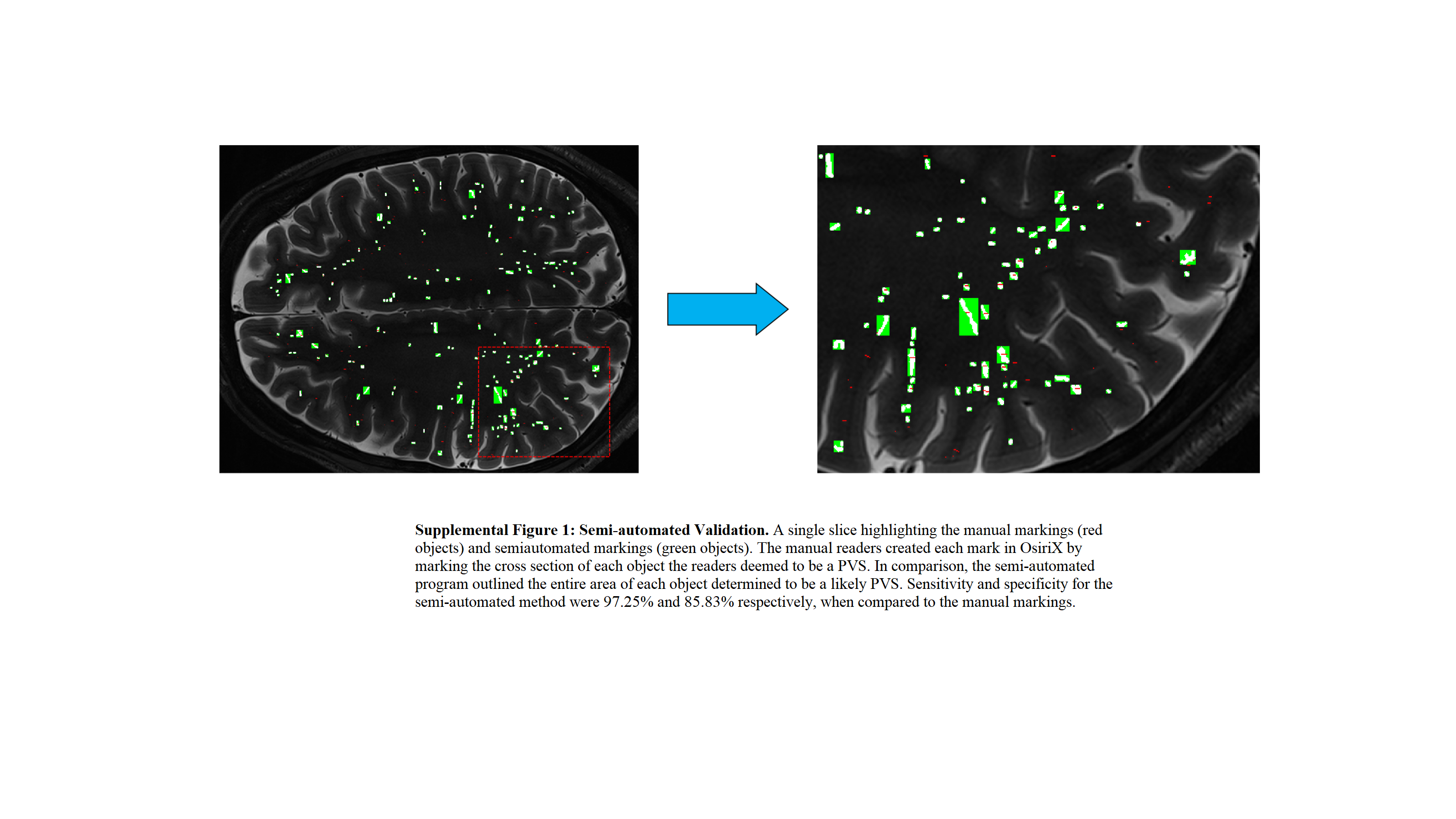

Supplement: Supplementary file 1 — Supplemental Figure 1: Semi‐automated Validation. A single slice highlighting the manual markings (red objects) and semiautomated markings (green objects). The manual readers created each mark in OsiriX by marking the cross section of each object the readers deemed to be a PVS. In comparison, the semi‐automated program outlined the entire area of each object determined to be a likely PVS. Sensitivity and specificity for the semi‐automated method were 82.9% and 91.9% respectively, when compared to the manual markings. [file BRB3-12-32598-s001.png]
